# Strain tunable magnetism in SnX_**2**_ (X = S, Se) monolayers by hole doping

**DOI:** 10.1038/srep39218

**Published:** 2016-12-19

**Authors:** Hui Xiang, Bo Xu, Yidong Xia, Jiang Yin, Zhiguo Liu

**Affiliations:** 1 National Laboratory of Solid State Microstructures and Department of Materials Science and Engineering, Nanjing University, Nanjing, 210093, China; 2 Collaborative Innovation Center of Advanced Microstructures, Nanjing University, Nanjing, 210093, China

## Abstract

By first-principles calculations, the magnetism of hole doped tin dichalcogenides SnX_2_ (X = S, Se) monolayers is systematically studied. It is found that a phase transition from nonmagnetic to ferromagnetic ground state appears once above the critical hole density (~10^14^ cm^−2^). The spin magnetic moment can maintain a magnitude of 1.0 *μ*_*B*_/hole with excellent stability of ferromagnetic state. Furthermore, we demonstrate that strain is very useful to modulate the DOS near the valence band, resulting in the reduction of the critical hole density to ~10^13^ cm^−2^ when the strain reaches 4% (6%) in SnS_2_ (SnSe_2_), which can be realized in common field effect transistors. Moreover, the phonon dispersion calculations for the strained SnX_2_ monolayers indicate that they can keep the dynamical stability under the hole doping. Therefore, the strain tunable magnetic transition in hole doped tin dichalcogenides indicates their potential promising applications in spintronic devices.

Atomically thick two-dimensional (2D) layered materials are currently one of most research interests for their promising applications in electronics, optoelectronics and spintronics^1^,^2^,^3^,^4^. By introducing local magnetic moments, many 2D systems have been proven to be important candidates in spintronic devices, such as spin field effect transistors (FETs), spin light-emitting diodes (LEDs) and solid-state quantum information processing devices^5^,^6^,^7^,^8^,^9^. But 2D semiconductors are naturally nonmagnetic, thereby it is a challenge to generate the stable magnetism. Previous studies have demonstrated that doping 3*d* transition-metal (TM) ions or 4*f* rare-earth metal ions into semiconductors were an achievable approach to induce magnetism^5^,^10^,^11^. But the extrinsic ferromagnetism induced by unpaired *d* or *f* electrons were undesirable for practical applications, owning to their limiting transports of spin polarized carriers.

In addition to the magnetic elements doping in semiconductors, the type of *d*^0^ ferromagnetic semiconductors^12^,^13^,^14^, the absence of atoms with partially filled *d* or *f* bands, has received considerable attentions in recent years, due to their intrinsic spontaneous magnetization based on *sp* states of nonmagnetic atoms. Moreover, these systems indicated obvious advantages in strong long-range exchange coupling interaction and no clustering of magnetic ions. To obtain 2D *d*^0^ ferromagnetic semiconductors, most studies have focused on inducing local magnetic moment by introducing nonmagnetic impurity atoms^15^,^16^,^17^, vacancies^18^,^19^, as well as manipulating nanoribbon edges^20^,^21^,^22^. In the band-picture model, the spontaneous magnetization in these *d*^0^ semiconductors occurs when the relative gain in exchange interaction is larger than the loss in kinetic energy, i.e., when it satisfies the “Stoner Criterion”: *D*(*E*_*F*_)*J* > 1, where *D*(*E*_*F*_) is the density of states (DOS) at the Fermi level (*E*_*F*_), and *J* denotes the strength of the exchange interaction^23^. Studies have indicated that *J* is large enough in elements N, O, P and S valence *p* orbitals^12^. When the *D*(*E*_*F*_) becomes large enough to satisfy the Stoner Criterion, systems can be spin polarized. So the prime purpose of chemical doping and other methods is to increase the DOS at *E*_*F*_. However, these approaches would face serious challenges in experiments, for the structural disorder inherently always causes unnecessary complexity in the physical properties, even the concentration of dopants and vacancies is unfavorable for controlling.

It is worth noting that some 2D semiconductors exist unusual band structures, one of which is the Mexican hat dispersions, such as gallium or indium monochalcogenides, often resulting in van Hove singularities (VHSs) with 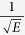 divergence in the DOS^24^,^25^. Once the *E*_*F*_ locates near the top of valence band, the high DOS generally leads an electronic instability, resulting in phase transitions such as magnetism, superconductivity, and other phenomena. In semiconductor industry, carrier doping is considered as an effective approach to modulate *E*_*F*_, which performs obvious advantages including their completely free of structure disorder and remarkably simple to tune physical properties^26^, compared to previously mentioned approaches. By hole doping in 2D systems, Cao *et al*.^27^ reported the theoretical investigation of magnetism in monolayer GaSe, and Huang *et al*.^28^ demonstrated a robust half-metallic spin-polarized state in silion phosphides (C2/m Si_1_P_3_). In the previous work, we also explored the hole doping effect on magnetic properties in 2D graphene-like C_2_N^29^.

In recent years, chemically stable and environmentally friendly semiconducting tin dichalcogenides SnX_2_ (X = S, Se) have been widely studied for their excellent electrical, optical and magnetic properties, such as lithium ion batteries, photovoltaic devices, as well as field effect transistors^30^,^31^,^32^,^33^. These studies generally indicated the unusual electronic structure with the high DOS and small dispersion near the top of valence band, which would provide a probability for magnetic phase transition. Although several calculations have demonstrated the magnetism in SnX_2_ nanostructures, such as manipulating SnSe_2_ armchair nanoribbons via edge hydrogenation^22^ and doping metal elements (Li, Mg and Al) in single-layer SnS_2_^34^, the disadvantage of which would affect their performance in further experiments for their unexpectable complexity and uncontrollability. In this work, by high density hole doping, we mainly study the magnetic characteristic of pristine and strained SnX_2_ (X = S, Se) monolayers. Results indicate that ferromagnetic ground state with outstanding stability of spin polarization can be obtained by hole doping. Particularly, by strain engineering, the critical hole density can be reduced to 10^13^ cm^−2^, which is an order of magnitude smaller than that of pristine structure. Therefore, SnX_2_ monolayers can be considered as a viable candidates for spintronic devices.

## Computational method

To study the electronic and magnetic properties of the SnX_2_ monolayers, density functional theory (DFT) calculations were performed using the Projector-Augmented Wave (PAW) pseudopotential implementation of the Vienna Ab Initio Simulation Package (VASP)^35^,^36^,^37^. Electron exchange and correlation effects were described by the generalized gradient approximation (GGA) functional of Perdew-Burke-Ernzerhofer (PBE) formula^38^. The energy cutoff for the plane-wave basis was set as 550 eV on the 11 × 11 × 1 Monkhorst-Pack *k*-point grid for all simulations. The convergence threshold was 1 × 10^−5^ eV for the electronic self-consistent field iterations. The atomic positions were optimized until the maximum Hellma-Feynman force on each atom was less than 10^−2^ eV Å^−1^. A vacuum spacing of 20 Å was placed to avoid the interactions between the monolayers and its periodic images. Moreover, to examine the dynamical stability of SnX_2_ monolayers with doping, the phonon dispersion was calculated by density functional perturbation theory in VASP.

## Results and Discussion

**Figure 1:**
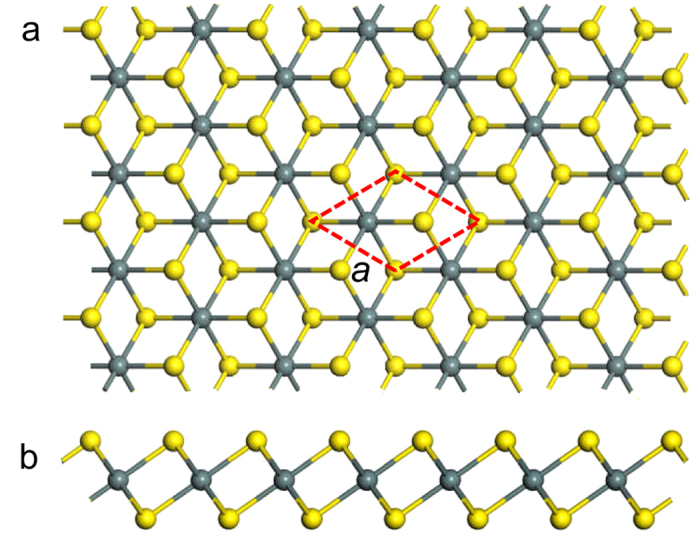
The top view (**a**) and side view (**b**) of SnX_2_ (X = S, Se) monolayers. The yellow and green balls represent X and Sn atoms, respectively. The rhombus with red dashed line represent the primitive unit cell, which is also the computation unit cell.

SnX_2_ (X = S, Se) monolayers with the 1*T* structure are the hexagonal crystal structure of the CdI_2_-type, as shown in Fig. 1. The SnX_2_ monolayers consist of three atomic sublayers with the covalently bonded layer of X-Sn-X. The central Sn atom bonds to six nearest-neighbor X atoms located in the top and bottom sublayers. The structural stability of monolayer SnS_2_ has been theoretically predicted by Zhuang *et al*.^32^ For all simulations, the primitive rhombic unit cell was used as the computation unit cell. Before investigating the electronic and magnetic properties, geometric structures of SnX_2_ were optimized, and atomic positions were relaxed to zero pressure following the convergence criteria. The optimized lattice constant of SnS_2_ and SnSe_2_ is 3.700 Å and 3.871 Å, which is in accordance with previous calculations by PBE formula^32^,^33^,^39^.

**Figure 2:**
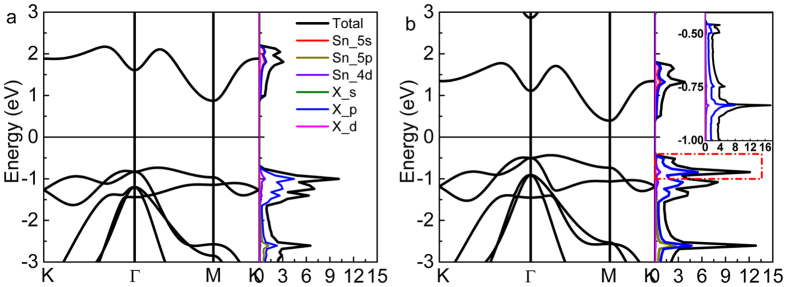
Calculated band structure (left) and the total and partial DOS (right) of monolayer SnS_2_ (**a**) and SnSe_2_ (**b**). The inset map in (**b**) is the detailed total and partial DOS of SnSe_2_ near the VBM with the small region of −1.0 to −0.4 eV, as marked in the red dashed rectangle.

The band structure and DOS of SnX_2_ monolayers are displayed in Fig. 2. It is obvious that the conduction band minimum (CBM) is located at the *M* point and the valance band maximum (VBM) lies between *Γ* − *M* point, which means both the SnS_2_ and SnSe_2_ monolayers are indirect band gap semiconductors. The indirect band gap of SnS_2_ (~1.60 eV) is almost double of SnSe_2_ (~ 0.83 eV), which is consistent with previous reported value (1.57 eV for SnS_2_ and 0.79 eV for SnSe_2_)^39^ by the same method. Due to the underestimation by PBE formula, the band gap is smaller than that of previous reports by simulations with Heyd-Scuseria-Ernzerhof (HSE) hybrid functional calculations and quasiparticle self-consistent GW methods^32^. In order to analyze the contribution of each orbit of atom, the total DOS, together with the 5*s*, 5*p*, 4*d*-orbit partial DOS (PDOS) of Sn atom, and *s, p*-orbit PDOS of S (Se) atom are depicted in the right diagrams of Fig. 2. It is clear that the high DOS is located around the VBM, which is mainly contributed by S-3*p* (Se-4*p*) states, besides, the CBM is co-contributed by Sn-5*s* and S (Se)-*p* states.

**Figure 3:**
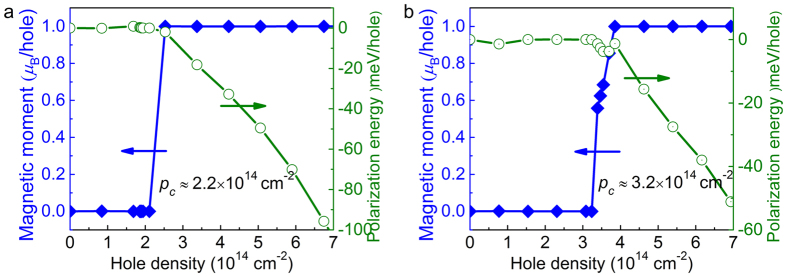
The magnetic moment and spin polarization energy of monolayer SnS_2_ (**a**) and SnSe_2_ (**b**), where the blue solid rhombus represent the relation between the magnetic moment and the hole density, and green hollow circle represent the relation between the spin polarization energy and the hole density, respectively.

It is well known that the high DOS around the *E*_*F*_ would provide the possibility to develop a spontaneous ferromagnetism. We applied carrier doping to investigate the possible ferromagnetism in SnX_2_ monolayers. Figure 3 shows the local magnetic moment (*μ*_B_) per hole with the various hole density. At the nonmagnetic state, the magnetic moment is nearly zero. Once above the critical hole density, the local magnetic moment increases rapidly to a constant value with ~1.0 *μ*_B_/hole, implying the transition from nonmagnetic to ferromagnetic ground state. The critical hole density (*p*_*c*_) of SnS_2_ and SnSe_2_ is about 2.2 × 10^14^ cm^−2^ and 3.2 × 10^14^ cm^−2^, respectively. In order to check the stability of ferromagnetic state, the spin polarization energy per hole, Δ*E*_*p*_, is discussed, which is defined by the energy difference between the spin-polarized state (*E*_*sp*_) and non-spin-polarized state (*E*_*nonsp*_) for each hole, i.e., Δ*E*_*p*_ = (*E*_*sp*_ − *E*_*nonsp*_)/*number*(hole). The hole density dependence of spin polarization energy per hole is also shown in Fig. 3. At the spin polarized states, Δ*E*_*p*_ decreases monotonically with the increment of the hole density. At the hole density of 7.0 × 10^14^ cm^−2^, Δ*E*_*p*_ is about −103 and −52 meV/hole for SnS_2_ and SnSe_2_, respectively, much higher than those in GaSe (−3 meV/hole)^27^ and Si_1_P_3_ (−24 meV/hole)^28^, indicating that the ferromagnetic ground state in hole doped SnX_2_ would be much more stable.

**Figure 4:**
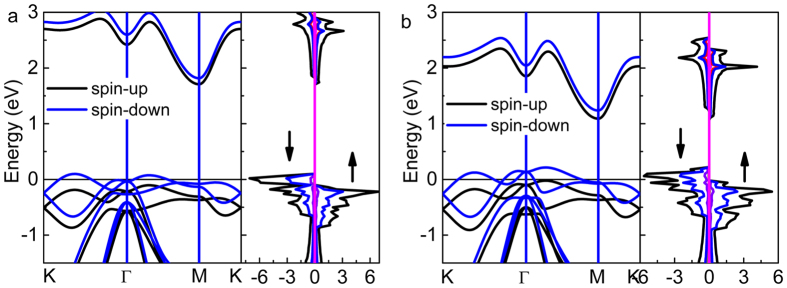
The typical band structure, and total and partial DOS of monolayer SnX_2_ in the spin polarized ferromagnetic state, with the hole density of 4.4 × 10^14^ cm^−2^ of SnS_2_ (**a**) and 5.4 × 10^14^ cm^−2^ of SnSe_2_ (**b**), respectively. The spin up (↑) and spin down (↓) electronic states are depicted in the right diagram.

We also studied the band structure and DOS of hole doped SnX_2_ monolayers. At the first stage, although hole doping leads to the *E*_*F*_ shift down to around the VBM, the spin up and spin down bands completely overlap, indicating the nonmagnetic structure. Once the hole density exceeds the critical value, the spin splitting appears around *E*_*F*_, resulting in the magnetic transition. When the hole density continue to increase, the spin up bands shift down, meanwhile, spin down bands shift up gradually. Figure 4 shows the typical electronic structures of hole doped SnX_2_ in the spin polarized state with the hole density of 4.2 × 10^14^ cm^−2^ for SnS_2_ and 5.4 × 10^14^ cm^−2^ for SnSe_2_. Moreover, it is found that the spin down channel shows metallic characteristic, while the spin up channel shows a fascinate transition from metal to semiconductor, where the transition occurs at 2.5 × 10^14^ cm^−2^ for SnS_2_ and 5.4 × 10^14^ cm^−2^ for SnSe_2_, respectively. Thus, the half-metallic SnX_2_ monolayers can be modulated by hole doping. This half-metallic property would have good performance in transferring one particular spin oriented electrons such as in a spin filtering devices.

To understand the magnetization mechanism of hole doped SnX_2_ monolayers, we notice that the magnetic moments are contributed mainly by the *p* states at the VBM. Like the previous study on *d*^0^ ferromagnetic semiconductors, such as ZnO^12^, GaSe^27^ and Si_*x*_P_*y*_^28^, the band-picture model also can be employed to explain the magnetization mechanism of hole doped SnX_2_ monolayers. The ferromagnetic characteristic would be achieved through a *p*-*d* hybridization-like *p*-*p* interaction between the *p* states of chalcogens. Figure S1 depicts the evolution of *D*(*E*_*F*_) and the energy difference *ΔE* of the two spin-type bands around the VBM by hole doping for monolayer SnX_2_. A large exchange-splitting of the two spin-type bands appears due to the strong exchange field in the ferromagnetic phase, and the *ΔE* increases with the increment of hole density when the *D*(*E*_*F*_) is large enough, which means a large strength of exchange interaction *J* in these systems. Consequently, the magnetism of SnX_2_ monolayers can be induced by hole doping.

Currently, ultrahigh carrier density accumulation could be achievable using electric-field control in FETs in experiments, including conventional solid state gated voltage in FETs and electric double layer transistors (EDLTs) by using polymer electrolytes or ionic liquid as gated dielectrics. By using EDLTs, an accumulation of extremely high carrier density could up to 10^14^ cm^−2^ in graphene and transitional metal dichalcogenides^40^,^41^,^42^, while ~10^13^ cm^−2^ for conventional solid state gated voltage in FETs^43^,^44^. But doping with high hole density often brings unexpected uncontrollability and difficulty, even by EDLTs. Therefore, it is crucial to reduce the hole density in SnX_2_ monolayers for their further practical applications.

The detail DOS of SnSe_2_ in the small region from −1.0 to −0.4 eV are inset in Fig. 2b. It is find that the high DOS near VBM is located at the deep level, which brings us an insight to analyze the reason why such high critical hole density (~10^14^ cm^−2^) must be needed to obtain magnetic transition. Therefore, we devote to modulating the high DOS to the top of valence band. As we known, band structure is highly sensitive to the external conditions such as temperature, pressure or strain, especially in 2D layered structure. They often lead to dramatically changes about electronic, magnetic and optical properties. In particular, the strain engineering is commonly used to tune the band structure, due to the extraordinary break strength and structural stability in a wide range of strain^45^,^46^,^47^,^48^,^49^. Zhou *et al*. recently reported the effects of in-plane biaxial strain on the electronic structure of single-layered SnS_2_^50^. They found that the tensile strain could result of a larger value of states around the Fermi level. So we believe that the tensile strain should be an effective way to reduce the critical hole density.

**Figure 5:**
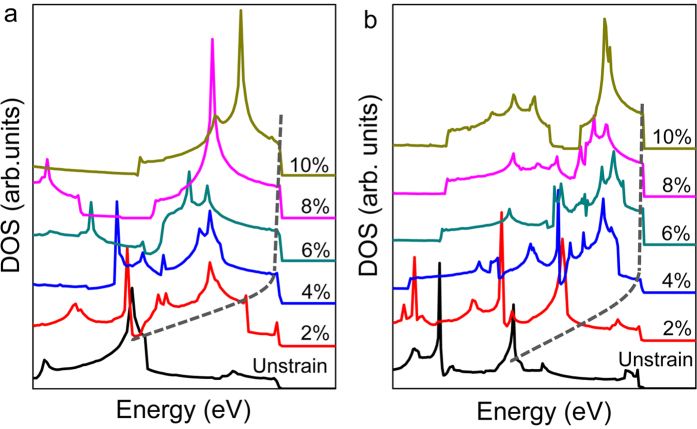
DOS of SnS_2_ (**a**) and SnSe_2_ (**b**) with biaxial strain from 2% to 10%, in addition with the unstrained state. The gray dashed lines indicate the location of the high DOS near VBM.

In this study, the biaxial tensile strain [2%, 10%], with the increment of 2%, was applied for the SnX_2_ monolayers. Figure 5 shows the evolution of DOS near valence band edges for strained SnX_2_ monolayers, in addition with unstrained states. It is clear that the DOS near the VBM increase with the increment of strain. When the strain reaches 4% for SnS_2_ and 6% for SnSe_2_, the DOS around the VBM are similar, and the VHSs occur, due to the Mexican hat-like band edges around *Γ* point. Once the *E*_*F*_ shift down to the top of valence band, such unusual DOS would lead to the reduction of the hole density, and transitions to magnetism. So our next work studies the local magnetic moment and spin polarization energy dependent on the hole density under various strain strength.

**Figure 6:**
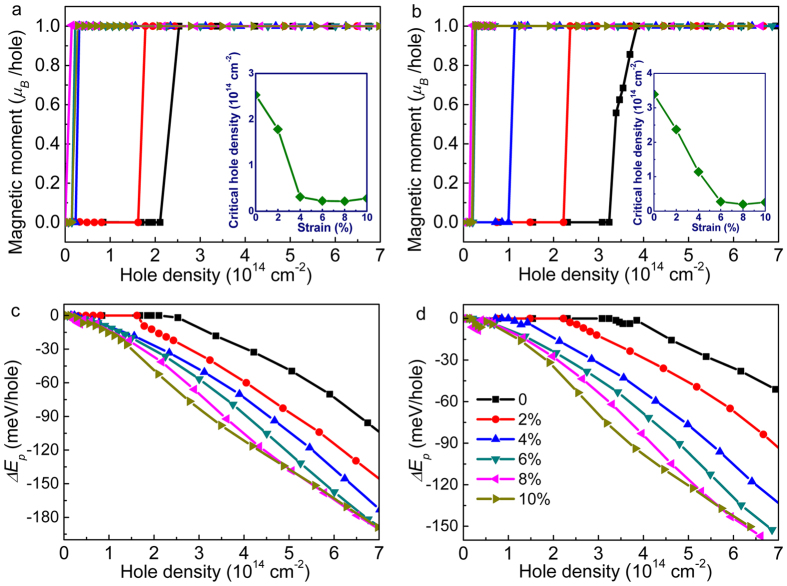
The magnetic moment/hole of SnS_2_ (**a**) and SnSe_2_ (**b**) with biaxial strain applied [2%, 10%] in the large range of 0~7.0 × 10^14^ cm^−2^, where the inset represent relations between the critical hole density and tensile strain. (**c**) and (**d**) Depict the spin polarization energy/hole of SnS_2_ and SnSe_2_, respectively. The magnetic moment/hole and spin polarization energy/hole of unstrained structure are also marked in (**a–d**) with black color.

The relations between the spin magnetic moment/hole and the hole density are shown in Fig. 6a and b. At the initial strained SnX_2_ monolayers without hole doping, it is nonmagnetic at the ground state. The magnetic moment (~1.0 *μ*_B_/hole) appears at the lower hole density in comparison to the unstrained SnX_2_. The inset map plotted relations between the critical hole density and the tensile strain. The critical hole densities reduce sharply in the small range of strain, i.e., 0–4% for SnS_2_ and 0–6% for SnSe_2_, and then maintain about 1.5 × 10^13^ cm^−2^ of SnS_2_ and 2.0 × 10^13^ cm^−2^ of SnSe_2_, respectively. These values are an order lower than that of unstrained structures, and could be realized in common FETs. Moreover, with the biaxial strain applied, the tendency of critical hole density is consist with that of DOS around the VBM described in Fig. 5. In addition, Fig. S1 also shows the *D*(*E*_*F*_) and *ΔE* for strained SnS_2_ (SnSe_2_) monolayers. It is clear that the SnX_2_ monolayers under biaxial strain are more favorable to achieve the spin splitting due to the greatly enhanced *D*(*E*_*F*_). Next, we check the stability of spin polarization of the strained structure, as shown in Fig. 6c and d. It is obvious that the spin polarization energy/hole reduce monotonously at the magnetic state in a large range of hole density. Compared to the unstrained SnX_2_ monolayers, the lower spin polarization energy/hole can be obtained with the increment of strain, and finally the spin polarization energy nearly tend to the constant value. For example, at the hole density of 7.0 × 10^14^ cm^−2^ with 6% strain applied, values decrease monotonically to −180 meV of SnS_2_ and −160 meV of SnSe_2_, which is far more small than that of the unstrained structure. Therefore, by applying tensile strain, the magnetism of SnX_2_ monolayers would have promising applications for their achievable hole doping density and stable magnetic phase transition.

In addition, the critical hole density, magnetic moment and spin polarization energy are summarized for SnX_2_ in Table 1, in comparison with GaSe^27^ and C2/m Si_1_P_3_^28^. The magnetism in unstrained structure requires higher hole density doping. But applying tensile strain (≥4% for SnS_2_ and ≥6% for SnSe_2_), the critical hole density of SnX_2_ monolayers is smaller that of Si_1_P_3_ and GaSe. Furthermore, SnX_2_ monolayers perform better stability of spin polarization under both unstrained and strained state. Therefore, for 2D SnX_2_ monolayers, hole doping can induce stable spin polarization in a large range of hole density, and the critical hole density and spin polarization energy can be improved greatly by strain engineering.

**Figure 7:**
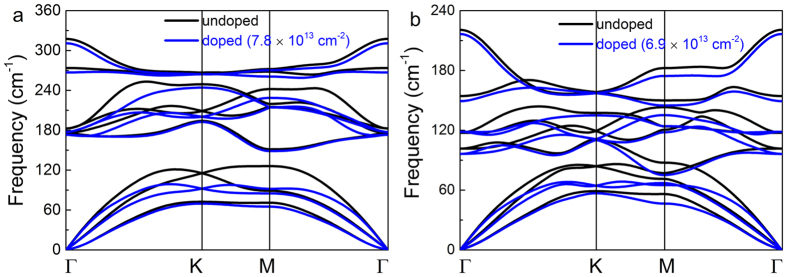
The phonon dispersion with 4% (6%) strain for SnS_2_ (SnSe_2_), where blue lines represent the hole density of 0.1 hole per unit cell, i.e., 7.8 × 10^13^ cm^−2^ for SnS_2_ and 6.9 × 10^13^ cm^−2^ for SnSe_2_. The results of undoped strained states are depicted in (**a**) and (**b**) with black color as well for comparison.

The stability of hole doped SnX_2_ monolayers is crucial for their applications on magnetism. So, we have also carried out the phonon dispersion calculations to study their dynamic stabilities. The phonon dispersions of SnS_2_ (SnSe_2_) monolayers under the tensile strain of 4% (6%) are shown in Fig. 7. No imaginary frequency is found under the tensile strain, indicating the SnS_2_ (SnSe_2_) monolayers under tensile strain are stable. At 0.1 hole per unit cell doping, large enough to induce magnetism in SnS_2_ (SnSe_2_) monolayer with tensile strain of 4% (6%), there is still no imaginary frequency, except the frequency softening comparing to the SnS_2_ (SnSe_2_) monolayers without doping. Therefore, these results indicate that hole doping in SnX_2_ monolayers have negligible effect on their structural stabilities, and will provide feasible theoretical predictions for practical applications in spintronic devices.

## Conclusions

In conclusion, electronic and magnetic properties of the SnX_2_ (X = S, Se) monolayers are studied by DFT calculations. It is demonstrated that hole doping (~10^14^ cm^−2^) can drive a phase transition from nonmagnetic to ferromagnetic ground state. The electron spin magnetic moment can reach a constant magnitude (~1.0 *μ*_B_/hole) with excellent stability of ferromagnetic state. Besides, the half-metallic characteristic can be modulated by hole doping. Furthermore, by introducing tensile biaxial strain to SnX_2_ monolayers, the critical hole density can reduce to ~10^13^ cm^−2^, which could be realized by conventional solid high dielectric FETs. The higher spin polarization energy indicates better stability in comparison with the pristine structure. In addition, the hole doped SnX_2_ monolayers still remains stable by phonon dispersion calculations. Therefore, investigation of the magnetism for hole doped SnX_2_ monolayers under the strain will provide an achievable idea for 2D functional materials in spintronics.

## Additional Information

**How to cite this article**: Xiang, H. *et al*. Strain tunable magnetism in SnX_2_ (X = S, Se) monolayers by hole doping. *Sci. Rep.* **6**, 39218; doi: 10.1038/srep39218 (2016).

**Publisher's note:** Springer Nature remains neutral with regard to jurisdictional claims in published maps and institutional affiliations.

## Supplementary Material

Supplementary Information

## Figures and Tables

**Table 1:** The critical hole density, magnetic moment and spin polarization energy for SnX_2_, GaSe, and C2/m Si_1_P_3_ are summarized, where *ΔE*_*p*_ of SnX_2_ gives the value at the hole density of 7.0 × 10^14^ cm^−2^.

	SnS_2_	SnSe_2_	SnS_2_ (4%)	SnSe_2_ (6%)	GaSe	Si_1_P_3_
Critical hole density (10^13^ cm^−2^)	22.4	32.3	1.5	2.0	3.0	9.0
Magnetic moment (*μ*_*B*_/hole)	1.0	1.0	1.0	1.0	0.9	0.98
*ΔE*_*p*_ (meV/hole)	−103	−52	−170	−160	−3	−23
