# Supplementary material for: Strain tunable magnetism in SnX2 (X = S, Se) monolayers by hole doping
Source: Sci Rep. 2016 Dec 19;6:39218. doi: 10.1038/srep39218 (PMC5171787; doi:10.1038/srep39218)
Supplement: Supplementary Information [file srep39218-s1.doc]

Supplementary Information

**Strain tunable magnetism in SnX_2_ (X = S, Se) monolayers by hole doping**

Hui Xiang^a^, Bo Xu^a,*^ , Yidong Xia^a^, Jiang Yin^a, b,*^ and Zhiguo Liu^a, b^

*^a^ National Laboratory of Solid State Microstructures and Department of Materials Science and Engineering, Nanjing University, Nanjing, 210093, China*

*^b^ Collaborative Innovation Center of Advanced Microstructures, Nanjing University, Nanjing, 210093, China*

^*^ Corresponding Author: [xubonju@gmail.com](mailto:xubonju@gmail.com) and [jyin@nju.edu.cn](mailto:jyin@nju.edu.cn).


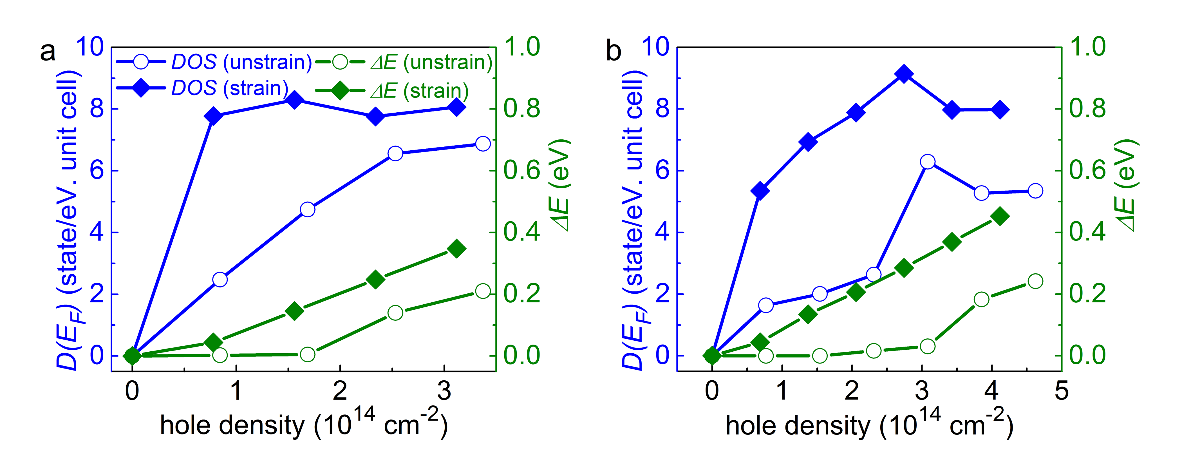


**Figure S1.** The evolution of *D*(*E_F_*) and the energy difference *ΔE* of the two spin-type bands around the VBM for monolayer SnS_2_ (a) and SnSe_2_ (b) by hole doping, where blue and green dotted lines represent the *D*(*E_F_*) and *ΔE*, and the hollow circles and solid rhombus represent the unstrained and strained (4% for SnS_2_ and 6% for SnSe_2_) structures, respectively.
